# Felt embodiment as a motive in flourishing

**DOI:** 10.3389/fpsyg.2025.1687553

**Published:** 2025-11-25

**Authors:** Lars-Gunnar Lundh, Lo Foster

**Affiliations:** Department of Psychology, Lund University, Lund, Sweden

**Keywords:** embodiment, embodiment motivation, disembodiment motivation, need, motive, flourishing, phenomenology, mindfulness

## Abstract

As individual persons, we are all embodied in the sense that we have a body with certain needs, sensory systems, feelings, and capacities. Humans and some other animals also have an *experience* of embodiment, defined as an experience of “*my* body.” This is a combined experience of *having* a body (the body as an object that can be perceived, imagined, thought about, and evaluated according to different standards) and *being this body* (the subjective experience of the *felt* body). The latter is not *about* the body as an object but is focused on *how* the body feels. The main claims of the present paper are (1) that we have a *need* to feel embodied, and that this is important for the development of self-identity, interpersonal relations, wellbeing, and flourishing in general; and (2) that experiences of feeling embodied can serve as important *motives*, both in a positive sense (embodiment motivation) and a negative sense (disembodiment motivation). In the present paper, felt embodiment motivation is illustrated by the motivation to feel one’s body (1) in its vitality and capacities, both in rest and in movement (e.g., physical activity and dancing), and in its expressiveness; (2) as being an integrated part of wholesome environments, and (3) in pleasurable and loving bodily contact with significant others. *Dis*embodiment motivation is defined as the motive to avoid or reduce bodily self-experiences, for example due to physical or emotional pain or other forms of bodily discomfort. In brief, the present paper outlines a new area of research: *embodiment motivation*, defined as the motivation to feel one’s own body, its vitality, capacities, and expressiveness, its embeddedness in wholesome environments, and the embodied contact with others, and its opposite: disembodiment motivation.

## 1 Introduction

As individual persons, we are all *embodied* in the sense that we have a body with certain needs and feelings, sensory systems that provide us with specific kinds of perspectives on our environment and on ourselves, and a motor apparatus that enables us to move around and interact with our surroundings in certain ways. As the other side of the coin, our specific forms of embodiment also involve various constraints in all these respects (e.g., as humans we cannot fly like a bird). Moreover, we have a subjective *experience* of our embodiment as moving, sensing individuals in interaction with the physical and social surroundings. In the present paper, it is further argued that we have a *need* to feel embodied, and that felt embodiment can have positive effects on subjective wellbeing and on mental and physical health. This means that felt embodiment represents a dimension of human *flourishing,* and that experiences of feeling embodied can serve as important *motives* for us. In brief, the present paper outlines a new area of research: *embodiment motivation*, defined as the motivation to feel one’s own body, its vitality, capacities, and expressiveness, its embeddedness in wholesome environments, and the embodied contact with others.

The paper is organized in four main sections. First, the basic conceptual framework is outlined, with a definition of the main concepts, such as “embodiment,” “felt embodiment,” “needs,” “motives,” and “flourishing” (section 2). Then we illustrate our basic reasoning concerning the needs and motives for felt embodiment by discussions of three different areas: felt embodiment in movement and physical activity (section 3), felt embodiment as part of wholesome environments (section 4), and felt embodiment in relation to other persons (section 5).

## 2 The conceptual framework: embodiment, experience, needs, and motives

It is important to differentiate between embodiment as such and *experienced* embodiment. Various writers have argued that mental processes are embodied in the brain (e.g., Edelman, 1989, 1992) and body (e.g., Thompson, 2007; Varela et al., 1991), whereas other writers (e.g., Al-Saji, 2010; Husserl, 1989; Sheets-Johnstone, 2020) have had their focus more on the *experience* of embodiment. Although the focus of this paper is on felt embodiment, the present section starts by placing experienced embodiment within the larger perspective on mental processes as embodied in the brain, body, and environment, before it proceeds to the main topic of *felt embodiment*.

### 2.1 The nature of embodiment

The nature of embodiment means that we are embodied creatures with a certain kind of brain, certain kinds of sensory systems, and a certain kind of motor apparatus. Altogether, these aspects of embodiment provide us with (1) specific *perspectives* on the surrounding world and on our own body, (2) an ability to *feel* our body from within, and (3) an ability to *move* around and interact with the physical and social environment—although, of course, there are functional variations. Individuals of different species are embodied in a wide variety of ways. An octopus, for example, is embodied in a very different way from human beings (e.g., Godfrey-Smith, 2017). There is also individual variation in embodiment within each species; among other things, human embodiment differs between the genders, and between individuals of different ages.

In addition to these synchronic aspects of embodiment, embodiment can also be characterized diachronically, as an individual’s life undergoes a developmental trajectory of bodily *changes* from birth to death. We arrive in the world as born from another human body (our mother). We grow and develop, we may be afflicted by various diseases and accidents and eventually recover from these, and finally we die. This means that embodiment can be seen, in a developmental perspective, as an aspect of human being that shows a life-long trajectory with typical themes and many variations.

All living individuals, from unicellular organisms to humans, are embodied, in the sense that they have a body with metabolism, homeostatic processes (Damasio, 2021), and processes of recursive self-maintenance (Bickhard, 2025). All animals have *needs*: for example, they need nourishment from the environment, and to keep the internal state of their body (e.g., its temperature) within certain limits (homeostasis), if they are to stay alive and prosper. For example, they need oxygen to breathe, food to eat, and water to drink, and they need to get rid of waste products by urination and defecation. They also need an ability to move their body away from dangers. All this illustrates how the individual’s particular kind of embodiment makes some things (e.g., food, water) and internal states (e.g., right temperature) *valued* over others.

Further, the evolution of animals with complex sensory organs and minds/brains have made them able to *perceive* what their environment *affords* (Gibson, 1966, 1979) in terms of what is valuable to their wellbeing. This development makes the individual animal able to *act* in relation to experienced *possibilities*, rather than merely responding to stimuli. Such experienced possibilities are basic to the development of *motives*. Motives, as defined here, represent *strivings to realize experienced possibilities*.

### 2.2 Experienced embodiment

Although individuals of many animal species obviously *feel* their body, as evidenced by their ability to experience pain, hunger, thirst, etc. (e.g., Denton, 2006; Panksepp, 2016; Thompson, 2022), it is more uncertain which species can conceive of their body as “*my* body.” Evidence of this kind of experience is seen, for example, in research findings with mirror-recognition tests which show that chimpanzees can recognize themselves in a mirror. In a test of this kind, the animal is typically anesthetized and then marked (e.g., with paint) on an area of the body (e.g., the forehead). After recovery from the anesthetic, the animal is given access to a mirror; if it then touches or investigates the mark on itself, this is taken to indicate that the animal perceives the reflected image as an image of itself (e.g., Gallup and Anderson, 2020). Although the sensitivity of this test may be questioned when it comes to some animals, the importance of this test here is that it illustrates what it means to experience one’s body as an observable “object” and not only as a subjectively felt body.

As understood here, the experience of embodiment involves the combined experience of these two aspects of bodily self-experience: *having* a body and *being this body.* In our previous work (Lundh and Foster, 2024), we have referred to the experience of *my body* as the *embodiment synthesis*. More specifically, the embodiment synthesis illustrates how we have two complementary perspectives on our own body:

- the body as an *object* that can be perceived (e.g., in the mirror), imagined, thought about, and evaluated according to different standards, and that can be used to act on our environment, and is also affected by the environment
- the subjective experience of the *felt* body, and how it feels to be a body of *this* specific kind interacting with the environment in *these* specific ways.

Whereas the former “objective” aspects are typically focused in research on body perception, body image and body esteem, the latter more “subjective” aspect is especially focused in research on *felt embodiment*.^1^ Moreover, although the embodiment synthesis is originally established as a *passive* synthesis in Husserl’s sense^2^—that is, it occurs spontaneously without any involvement of cognitive processes—it can be *actively* elaborated in different ways. Individuals may, for example, differ in how attentive they are to how their body is viewed by others (“the body I have”). Similarly, individuals may differ in how attentive they are to the felt body (“the body I am”). This means that, on top of the basic *passive* synthesis of embodiment, individuals are engaged in a life-long continuous *active* synthesis of embodiment—the development of their idiosyncratic experience of “my body.” Although we have previously (Lundh and Foster, 2024) described the important role of *attention* in this process, we said nothing about *motivational* processes. In the present paper, the focus is on motivation, and especially on the motivation to *feel* one’s own body (i.e., the subjective aspect of embodiment).

### 2.3 The nature of felt embodiment

Felt embodiment essentially involves subjective sensations and feelings. A central theme in Husserl’s analysis of bodily experience (Husserl,1989) is that the body differs from all other things by the fact that we *feel it from within*. In Husserl’s terminology, this takes the form of bodily *sensings*^3^. Although much of Husserl’s analyses in this area are focused on localized touch-sensations, he describes sensings as a wide category of feelings, of which some permeate and fill the entire body, such as pleasure and pain, the sense of wellbeing, feelings of general malaise, and “all kinds of sensations… that form the substrate for the life of desire and will, sensations of energetic tension and relaxation, sensations of inner restraint, paralysis, liberation, etc.” (Husserl, 1989, p. 160). Here we summarize these experiences under the concept of the *felt body*.

Damasio (2012) speaks about *primordial feelings* in this context. Primordial feelings are said to.

occur spontaneously and continuously whenever one is awake. They provide a direct experience of one’s own living body, wordless, unadorned, and connected to nothing but sheer existence. These *primordial feelings* reflect the current state of the body along varied dimensions, for example, along the scale that ranges from pleasure to pain, and they originate at the level of the brain stem rather than the cerebral cortex (Damasio, 2012, p. 21).

In Damasio’s view, feelings constitute *the subjective element of all kinds of emotions*: “[a]ll feelings of emotions are complex musical variations on primordial feelings” (Damasio, 2012 p. 21). Importantly, such feelings need not involve mental representations of some kind of “objects” that are separate from the experience itself. What is at stake is rather an experience of being alive, of “sheer existence” as Damasio (2012), p. 21 puts it. Conversely, this also means that our very experience of *being* always involves feelings: “There is no *being*, in the proper sense of the term, without a spontaneous mental experience of life, a feeling of existence” (Damasio, 2018, p. 100).

Other writers speak about such feelings in terms of “felt life” (Langer, 1967). As argued by Langer, these feelings form an intricate dynamic pattern of tremendous complexity, where our words for feelings only represent crude designations. What eludes the power of language, according to Langer, is “the way feelings, emotions, and all other subjective experiences come and go—their rise and growth, their intricate synthesis that gives our inner life unity and personal identity” (Langer, 1957, p. 7). Vendrell Ferran (2021) describes feelings of vitality in a similar way “as a broad range of experiences in which we feel the powers of life, its increments and decrements, its ups and downs” (p. 116). Examples are feeling energetic or dispirited, vigorous or weary, full of life or exhausted. In extreme fatigue, in illness, and at the end of life, we can feel “how life fades away from us,” whereas in excitement and happiness “we feel life pulsing through us” (p. 116). As she also notes, [w]henever we experience a feeling of vitality, we have an experience of our*selves*” (p. 125).

Importantly, this aspect of bodily experience, whether it takes the form of localizable body sensations, holistic bodily feelings or feelings of vitality, is not *about* the body as an object but is characterized by its focus on *how* the body feels from a first-person perspective. This makes it impossible to speak of sensations in the same objectifying way as we speak about external things. As Al-Saji (2000) puts it, “there are no single sensations, but systems and fields of sense” (p. 53); here she cites Husserl (1989), who spoke of *sensation-fields* and argued that the occurrence of new sensations takes the form of changes in the sensation-field.

Even if bodily sensations cannot be singled out as separable “objects,” however, we may clearly *shift attention* between different aspects of the sensation field (e.g., between sensations in different parts of the body, and between these and more holistic experiences of the body as a whole). This means that different sensations can stand out as being in focus at least momentarily. Moreover, this kind of bodily awareness can be trained by means of various practices such as yoga (e.g., Kee, 2023) and the so-called “body scan” as used in mindfulness programs (e.g., Kabat-Zinn, 2013; Shapiro and Carlson, 2017), where the individual systematically shifts their attention between different parts of the body.

This aspect of our bodily feelings is often neglected in the literature. Even in some phenomenologically inspired literature, the subjective feelings of bodily self-experience are relatively ignored, *except* when they occur as negative or unpleasant feelings. For example, when the body *does* come into focus of our experience, according to Fuchs (2022), it “turns into a physical, objective body” (p. 110) and this occurs “particularly when we become aware of it in a disturbing way, e.g., in fatigue, clumsiness, injury or illness” (Fuchs, 2022, p. 110). When the body does come into focus, in Fuchs’ perspective, it is as an *object*, and in a *disturbing* way, not as a *subjectively felt* and potentially *pleasant* bodily experience. Another example of this kind of thinking is Maiese (2024) reduction of the body-as-subject to “that which does the perceiving” (p. 774), with no mention of the possibility of attending to this “body-as-a-subject” as a *felt body*. In their kind of reasoning, the body-as-subject is basically reduced to an instrument or “medium” for our attention to external things, and there is no mention of the possibility of *focusing on* the “body-subject” as such, *without objectifying it*. In our perspective, however, it is essential that bodily feelings can be focused without turning them into objects, as qualitative aspects of *how it feels* to be not only in disharmonious but also in *harmonious* contact with one’s own body.

### 2.4 Needs, motives, values, and flourishing

It is important to distinguish between *needs* and *motives*. For example, we all have biological needs of various nutrients, but our eating is often motivated by how things taste rather than by their nutrition value. Often our motives *serve* our biological needs. But sometimes they come into conflict. For example, individuals with anorexia nervosa may even starve themselves to death; here the individual person’s motivation clearly does not serve their biological needs. This points to the essential distinction that is at stake here: *needs* are objective in the sense that they exist whether they are experienced or not, whereas *motives* are essentially experiential.

Basic needs represent an essential part of human embodiment. To stay alive, we need air to breathe, food to eat, water to drink, etc. But life is not only about *survival*. Individual lives may also differ as to their degree and quality of *flourishing*. Some lives end with an early death and are not allowed to flourish, whereas other lives are characterized by illness or various forms of suffering that allow only limited degrees of flourishing. As defined here, the concept of flourishing refers to wellbeing, mental and physical health, satisfying interpersonal relationships, some degree of realization of one’s talents, and other values that, although they are not needed for simple survival, are needed to ensure at least some degree of *life quality*.

Anything that promotes survival has *value* for the organism, and anything that promotes flourishing similarly has *value*. To ask what human beings need to flourish, however, is a more complex and difficult question than asking merely about what they need to survive. Several researchers have suggested taxonomies of human needs that are important both for survival and for flourishing. One of the first taxonomies was formulated by Murray (1938), who differentiated between essential bodily (“viscerogenic”) needs and psychosocial (“psychogenic”) needs, with the latter seen as fundamental although not biologically essential to human life. The most famous taxonomy is probably Maslow’s hierarchy of needs, which starts from basic physiological needs (Maslow, 1954), continues to successively higher levels of safety needs, needs for love and belongingness, and need for esteem, until it ends at the highest level as a need for self-actualization. There are, however, also theories that focus specifically on flourishing-related needs. One example is Deci and Ryan’s model of three basic needs (Deci and Ryan, 2000): competence (feeling effective in ongoing interactions with the social environment), relatedness (feeling connected to others), and autonomy (perceiving oneself as the origin or source of one’s own actions). Another example is Ryff’s model of six dimensions of wellbeing (Ryff, 1989): Self-acceptance, Purpose in life, Environmental Mastery, Personal growth, Positive relations with others, and Autonomy.

Common to all these kinds of needs, whether they are important for survival or “merely” for flourishing, is that they are assumed to exist whether we experience them or not. To the extent that we experience them, they can also serve as *motives* for us, and their strength may vary both across individuals and situations. One approach with this kind of focus is Reiss (2004) taxonomy of 16 fundamental motives, and his Profile of Motivation Sensitivity which is designed to measure individual differences in the strength of these motives. A basic assumption is that “individual variations in the strength of these motives are important for understanding a person’s life goals and everyday behavior” (Reiss and Havercamp, 1998, p. 98). A partly similar focus on individual differences in the prioritizing of different aspects of wellbeing is seen in the OECD Better Life Index; here the empirical results show considerable differences not only between individuals, but also between countries (e.g., Schleim, 2014).

The distinction between needs and motives is applicable also to embodiment. It is one thing to ask about people’s *need to feel embodied*, and it is another thing to ask about their *motives to feel embodied*. Evidence for a *need* to feel embodied is found if it can be shown that an increase in felt embodiment leads to improved health or wellbeing, and/or if a deprivation of felt embodiment leads to decreased health and wellbeing. Such effects may be searched for not only in the subjective experience of wellbeing but also in physiological signs of health or ill-health. Damasio (2021) has argued that bodily feelings are a kind of hybrids, “at once mental and physical” (p. 191), and that feelings are “commingled with the things and events we feel thanks to the exceptional and intimate cross talk between body structures and nervous system” (p. 71). Physiologically, he assumes that this is based on a *two-way interaction* between the nervous system and biochemical processes in the visceral part of our body, so that bodily changes are directly felt *and* that mental processes can have direct effects on physical states of the body.

Evidence that felt embodiment serves as a *motive* requires data showing that individuals engage in various activities *for the purpose of* feeling embodied. One example (Lundh, 2022b) is a patient who suffered from poor sleep who was instructed to use a Mindful Embodiment Practice (MEP) when having difficulties to fall asleep. The MEP is a short version of the body scan as used in mindfulness meditation programs, where the self-instruction is to attend to various parts of the body, exploring how it feels in each body part and then “breathe into” those bodily sensations. In this case, the patient not only enjoyed the practice but also felt *intrinsically motivated* to continue with the practice after treatment and even transferred it to other contexts with considerable effects on wellbeing. This illustrates one form of felt embodiment motivation.

If felt embodiment can have effects on wellbeing and health, it means that the motive to feel embodied can have a role in human *flourishing*. Conversely, it also means that the absence of such motivation, as seen either in a mere disregard of bodily feelings or in an active avoidance or suppression of bodily feelings, might lead not only to a loss of both wellbeing and health. A similar emphasis on the value of cultivating somatic sentience is found in Shusterman’s somaesthetics (Shusterman, 2008).

### 2.5 Variations of a thought experiment

Consider the following thought experiment on felt embodiment: Suppose you are offered to have part of your brain replaced by a very efficient computer that would allow you to *register all kinds of information* both about the surrounding world and about your internal bodily conditions (which would amount to a vastly improved interoception) in a way that would *far surpass your present cognitive abilities,* so that you could make fast and well-grounded decisions about how to act in a way that would increase your capacity for adaptation and survival. Suppose, however, that this would be *at the expense of* (1) your ability to *feel* your own body, including all forms of pleasure and pain, as well as (2) all *bodily* aspects of emotions such as joy, sadness, hope, fear, love and hate (i.e., while keeping and even improving your *cognitive* capacity to appraise and evaluate what happens to you in terms of gains and losses of things that are important for your survival and adaptation to the environment). To the extent that this loss of bodily experience would even make you hesitate to accept the deal that is offered, it shows that *felt embodiment* is one of your motives in life.

Now let us ponder the converse question: What would it take for someone to consider *accepting* the offer described in the previous paragraph? What about a person who suffers from unbearable pain and has little or no experience of positive feelings, and who therefore despairs that life is worth living? If the loss of bodily self-experience does appear to be a better alternative than suicide for that person, this can be said to exemplify a form of *disembodiment motivation*. Disembodiment motivation may be defined as the motive to reduce or even eliminate bodily feelings and sensations.

The *strength* of felt embodiment motivation (i.e., the motive to feel embodied) probably differs from one person to another, and it is an empirical question whether it is completely absent from anyone. The same, however, can also be said about *dis*embodiment motivation: it is an empirical question whether it is completely absent from anyone. All of us have probably experienced pain (physical or emotional) or other forms of bodily discomfort that we would rather be without. If this served as a motive for us to engage in some form of avoidance or suppression of feelings, this would be an example of at least a weak form of disembodiment motivation. Although strong forms of disembodiment motivation may be expected from people who suffer from severe forms of physical and emotional pain and trauma, some degree of disembodiment motivation may also be culturally conditioned. As Kee (2023) puts it,

the enculturation of bodies requires that we be able to silence and repress our bodily sensations and desires so that we can participate normally in the human cultural world. In more extreme though all too common cases, shame and trauma can make one’s own body a place of aversion or even terror. The upshot is that many of us end up living in a strained or complacent ignorance of our own bodies in our day-to-day adult life (Kee, 2023, p.138).

The concept of disembodiment motivation suggests an approach to problematic deficiencies in emotional awareness that differs from approaches which phrase the problems in terms of an inability to put *words* on one’s feelings. The latter kind of approach is perhaps best illustrated by the concept of alexithymia, which literally means “having no words for feelings.” The notion of disembodiment motivation suggests that problems with emotional awareness may go deeper than the ability to express one’s feelings in words, and that some forms of psychopathology may involve a motivation *not to* attend to bodily feelings in general. As Ferrarello (2024) has argued, the difficulties of finding words for one’s emotions may, in fact, be “primarily connected to the physical inability to ‘be there’ with the affect, and only secondarily with a representational problem” (p. 545). In other words, the problem may be an inability to stay in contact with the bodily experience of difficult emotions. Moreover, the problem may be wider than involving merely emotions; it may, in fact, involve a tendency to turn one’s attention away from bodily feelings in general, including feelings of vitality and exhaustion, as well as pleasure and pain.

### 2.6 Summary of section 2

- All living organisms are *embodied*, in the sense that they have a body with metabolism and bodily needs that must be satisfied if they are to stay alive and flourish.
- Humans and some other animals also have an *experience* of embodiment, defined as an experience of “*my* body.” This is a combined experience of *having* a body (the body as an object that can be perceived, imagined, thought about, and evaluated according to different standards) and *being this body* (the subjective experience of the *felt* body). The latter is not *about* the body as an object but is focused on *how* the body feels.
- It is important to distinguish between needs and motives. Evidence for a *need* to feel embodied requires data showing that felt embodiment leads to improved health and wellbeing, and/or that the deprivation of felt embodiment leads to decreased health and wellbeing. Evidence that felt embodiment serves as a *motive* requires data showing that people engage in various activities *for the purpose of* feeling embodied.
- The experience of felt embodiment, and the motive to feel embodied, differ across individuals and situations. In addition, people may experience *disembodiment* motivation—that is, they may be motivated to avoid or suppress bodily feelings—this motive may also differ from one person to another, and from one area of experience to another.

## 3 Felt embodiment in movement and physical activity

The purpose of this section is to discuss the importance of felt embodiment in connection with movement. Apart from movement in general, the focus is on two specific kinds of movement: physical exercise and dancing. The argument proceeds in three steps. First, we argue that movement is intrinsically connected with feeling—that is, movement is typically *felt* as movement. Second, we argue that we have a *need* to feel embodied in our movements. And third, we argue that felt movement can be an important *motive* in personal development.

### 3.1 The felt qualities of movement

Feelings, as Damasio (2012), p. 21 describes it, “occur spontaneously and continuously whenever one is awake” and thereby provide a direct existential experience of one’s own living body. This means that feelings are ubiquitously present also when we move. Although Damasio does not discuss felt movement in any detail, he notes that the role of feeling has been neglected by researchers in many areas of life. This also applies to research on movement.

The phenomenological tradition is an exception to this neglect; here movement and felt movement has long been in focus, from Husserl and onwards. Also, when Husserl (1970) speaks about *kinaesthetic consciousness* as a bodily “I can,” he includes not only the ability to feel the movements of our limbs and body, but also the ability to experience movement as a practical *possibility* even without performing it. Behnke’s term “kinaesthetic capability-consciousness” (Behnke, 2018) makes this very clear. What is at stake here is not only felt movement *in the act*, but also the ability to experience how a movement can feel even *before* (or even without) carrying it out. This also involves an experience of how *other* people’s movements may feel; that is, we do not perceive others’ movements simply as mechanical motion, but as impregnated with intention and feeling.

In a classical experiment, Heider and Simmel (1944) showed that if observers were presented with simple animated geometric shapes that were moving around on a screen, seemingly on their own accord (i.e., without being pushed into action by some external cause), the observers tended to perceive these geometric shapes as characters with emotions, intentions, and other experiences. This illustrates how certain patterns of movement tend to be directly perceived as expressive of feelings and intentions. Even though we do not confuse such geometric shapes with real persons, we have an almost irresistible tendency to experience their movements as expressive of feelings and intentions.

Sheets-Johnstone (2015, 2020) has written extensively about movement. She notes that we feel the *dynamic qualities* of our movements. Kinesthesia provides us with “a felt sense of the dynamics of our movement, its expansiveness, sluggishness, explosiveness, jaggedness, its changes in direction, intensity, range, and so on” (Sheets-Johnstone, 2015, p. 31). Different movements involve *distinctively felt dynamic patterns*. She illustrates this with children’s development of new movements, such as turning over, reaching, and crawling, and doing it slowly or rapidly:

The felt qualitative dynamics of movement in fact constitute distinctively felt patterns in each instance: what is dynamically felt in turning over is distinctively different from what is dynamically felt in reaching, just as what is later dynamically felt in crawling is distinctively different from what is later dynamically felt in walking. Moreover, what is specifically felt qualitatively in turning over rapidly, perhaps even suddenly, is distinctly different from what is specifically felt qualitatively in turning over slowly (Sheets-Johnstone, 2020, p. 6).

Just as in the case of the two complementary perspectives on our own body that were described in section 2.3 above (i.e., the body as an *object* of perception, imagination, and evaluation, and the subjective experience of the *felt body*), it is important to differentiate between two perspectives also in the experience of movement. As noted by Sheets-Johnstone (2020):

one may shift from experiencing a kinesthetically felt dynamics to experiencing a kinesthetically *perceived* object in motion, in effect no longer *feeling* a particular dynamic flow of movement, but *perceptually monitoring the flow* in some way. In doing so, one perceives reaching or reaching hand, for example, or crawling or crawling leg *objectively*, movement or body becoming an object in space and in time, a veritable *object in motion* (Sheets-Johnstone, 2020, p. 7).

We clearly have a need to *perceive* our movements if we are to monitor our movements in space and time. But do we also have a need to *feel* our movements? To ask this question is to ask if there are positive effects of felt movement on wellbeing, and/or negative consequences attached to the deprivation of felt movement.

### 3.2 The need to feel embodied in physical activity

Evidence clearly shows that we have a need for physical activity. In a review of this field of research, Stults-Kolehmainen (2023) concludes that bodily movement is important for proper circulation, oxygenation of tissues, metabolism, and many other functions. Although he concludes that deprivation of the need for physical movement is likely to produce unpleasant feelings of discomfort, tension and restlessness, as reported in terms of feeling “antsy” or “fidgety,” etc., however, this research has not much to say about the need to *feel our movements* when we engage in physical activity.

Most research on physical activity fails to take the *felt* experience of physical activity into account. Research on meditative movement practices such as Tai chi and Qigong, for example, shows evidence that such practices can have effects on heart rate variability (Larkey et al., 2024) and on symptoms of anxiety and depression (Lo et al., 2025), but this kind of research does not focus directly on the effects of *feeling* the movement. One approach to the study of *felt* experience is experimental phenomenology (Lundh, 2020, 2022a), where participants are instructed to engage in practices where they are to pay attention to their experiences with a certain attitude (e.g., mindful, accepting, explorative) to study the effects of such phenomenological practices.

In principle, all kinds of movements can be carried out with various degrees of mindfulness. An illustrative example is a study by Crum and Langer (2007), where room attendants were randomized into two conditions. Those in the experimental condition were told that their work (cleaning hotel rooms) is good exercise in line with an active lifestyle. At 4 weeks post-intervention the experimental group perceived themselves to be getting significantly more exercise than before, and showed an improvement on measures of physiological health as compared with the control group. The research questions, however, were formulated in terms of mindset rather than mindfulness, and the experimental design does not allow any conclusion whether the effects were due to an increased focus on the *felt* body (e.g., vitality feelings) or on the body as an *object* (e.g., an increased physical fitness); this would in principle be possible to explore with experimental-phenomenological designs.

One problem in research on felt movement and physical activity is how to label and categorize the various bodily feelings involved. According to some writers, such feelings largely “elude the power of language” (Langer, 1957). Two concepts that may be useful here are *vitality* and *expressiveness*. Vitality feelings may be defined as experiences of “feeling alive,” whereas feelings of expressiveness can be defined in terms of a fit between an inner experience and the outward movement.

Expressiveness as a form of embodiment motivation may be important in many areas of life. Expressive movement may be important not only for one’s individual wellbeing but also interpersonally and for the development of one’s self-identity. As argued by Husserl (1989), each individual has a personal style that is expressed in that person’s manner. To use an illustration from Heinämaa (2018): maleness and femaleness can be seen as two general types of bodily expressiveness with many different personal variants, which can either amplify or undo the male/female-duality.

Forlè (2019) argues that individuals develop a specific personal way of expressing their affective states in a way that allows their personality to emerge. This may be important not only as a form of self-presentation to others, but also for the development of the individual’s bodily felt *self*. Forlè (2019) suggests the hypothesis that the expressiveness of our movements is important for the development of our *self-identity*:

the expressive living body seems to be the dimension of our embodied nature in which the specificity of ourselves as ourselves—and as different from any other—can emerge. This is crucial not just for us to recognize other embodied beings as different individualized embodied *persons*, but also for us to recognize *ourselves* as the embodied persons we are (Forlè, 2019, p. 113).

As argued by Forlè (2019), “a personal stylistic mark can emerge” (p. 111) in the expressiveness of our felt body, so that it comes to express our *specific* and *personal* way of living these feelings. Moreover, this may occur in parallel with our grasping the specific expressive style of *others* in *their* bodily expressions, in a way that makes it possible to differentiate our own personal style from theirs. In other words, this points to the possible role of felt movements in the development both of our self-identity and our basic experience of others.

Felt movement may be important for the development of interpersonal relations. Roald et al. (2023) suggest that movement is a primary meaning-making modality, indispensable to the development of intersubjectivity. There is evidence to suggest that autistic children, even at an early age before being diagnosed with autism, show a relative absence of expressive movements, possibly due to disturbances in *kinaesthetic reafference,* which makes it difficult for them to fully sense their own bodies in motion. This relative absence of expressive movements may in turn create difficulties for parents to tune in to their children’s feelings, and for the child to sense and respond to the feelings of others:

if primordial feelings in autism are hampered by the disturbed ability to sense one’s own body in motion, so are the expressive features of primordial feelings, visible in muscle tone differences such as hypotonia and differences in movement kinematics and body postures. Consequently, such differences likely obstruct the autistic child’s developing ability to attune to the movements of others, and others’ ability to attune to the movements of the autistic child (Roald et al., 2023, p. 342).

Moreover, this may even create difficulties for the children to feel *themselves*, which might possibly explain their tendency to engage in repetitive forms of self-stimulation.

In much movement research movement is seen simply “as an object of experience rather than something intimately connected with subjective experience itself” (Roald et al., 2023, p. 346). From our perspective, it is important to note that, although we do perceive our movements (i.e., they are *objects* of our perception), we also sense them as part of our *felt* body. Moreover, this kind of *felt movement* may serve an important role in the development both of our intersubjective relations to others and for the development of our self-identity, and possibly also for wellbeing and physical health—it therefore seems fully reasonable to argue that we have a *need* to feel embodied in our movements.

### 3.3 Motives to feel embodied in physical activity: vitality and expressiveness

Some researchers have noted the importance of vitality feelings as a motive for engaging in physical exercise. Reiss (2004), for example, defines the motive for physical exercise as a desire to exercise one’s muscles while experiencing *intrinsic feelings of vitality* (p. 187). Interestingly, this formulation synthesizes a purpose related to the body as an *object* (the desire to exercise the muscles) with *subjective* body feelings (intrinsic feelings of vitality), thereby illustrating one variant of embodiment synthesis. Further, in a study of athleticism among college students, Reiss et al. (2001) conclude that the intrinsic enjoyment of physical exercise was the motive that was most strongly associated with participation in sports.

A limitation of the Reiss (2004) model, however, is that it fails to take account of the possibility that the relative strength of the motives (1) to exercise one’s muscles and (2) to enjoy the feelings of vitality may differ from one person to another. At one extreme, we may find individuals who are strongly motivated by the goal of strengthening their muscles, whereas the intrinsic enjoyment of the physical exercise plays little or no role—they would, in our terms, be high in “body-object motivation” and low in *felt* embodiment motivation. At the other extreme, we may find individuals who are strongly motivated by the intrinsic feelings of vitality that the physical exercise affords them, while caring little about the physical state of their muscles—they would, in our terms, be high in felt embodiment motivation and low in “body-object motivation.” And in between, we might find all kinds of combinations of these two motives. Here it is relevant to ask about the effects of different combinations of these two motives on wellbeing, and even on the actual tendency to engage in physical exercise. One hypothesis would be that the optimal motivation profile does not lie at any of the extreme ends of the spectrum, but somewhere in the middle.

Ryan and Frederick (1997) discuss subjective feelings of vitality and expressiveness in the context of physical activity:

One may expend energy on tasks that subjectively come from the self or on activities that one is compelled to do. A slave may be forced to build a stone tower, dragging stones from miles around. This tremendously effortful action is motivated and intentional, but would typically be experienced as draining vitality, since it detracts from one’s ability to behave autonomously and in ways that actualize or enhance the self. On the other hand, a sculptor who performs the same act of hauling and carving stone as a creative endeavor might feel vitalized or invigorated by this activity, as it emanates from and expresses the self. (p. 535).

However, although Ryan and Frederick (1997) refer to data showing associations between subjective vitality and psychological wellbeing, and claim that intrinsically motivated activity is “accompanied by feelings of vitality” (p. 534), they do not assign any *motivational* status to these feelings of vitality. Instead, they suggest the hypothesis that subjective vitality is “a function of conditions that support agency and growth” (p. 557); basically, they consider vitality feelings as merely a side-effect of behaving autonomously.

Again, however, we may ask about the relative importance of two different motives in the sculptor: (1) the goal of the creative activity, and (2) the feelings of vitality and expressiveness during the creative process. And in analogy with the example of physical exercise discussed above, it may be hypothesized that what is optimal for wellbeing and creativity is some kind of balance between these two motives. In fact, this perspective may be applied also to the Ryan and Frederick (1997) example of the slave who is “forced to build a stone tower, dragging stones from miles around.” Suppose some of the slaves, despite the almost unbearable hardships involved, do manage to enjoy some feelings of vitality in their physical work, thereby making it somewhat less unbearable. This could be seen as an illustration of Frankl’s thesis (Frankl, 1963) of the importance of finding some meaning even in the most dreadful situations imaginable, such as being a prisoner in a Nazi concentration camp, although the meaning in the present case would be found in the bodily feelings of vitality.

Feelings of vitality are not the only feelings experienced in connection with physical activity. Physical activity may also involve other bodily sensations and emotions—feelings of effort, tension, release, pain, joy, flow, pride, frustration, novelty, etc. It is an empirical question (1) which kinds of bodily sensations and emotions people feel in connection with physical activity, and (2) which of these are positively *valued* in such a way that they can *motivate* people to engage in physical activity.

### 3.4 Motives to feel embodied in dancing

Let us now consider the example of dancing. Here we may speak of an amalgam of motives, including not only (1) a *performance* motive, defined in terms of mastering a particular kind of dance with its specific steps and movements and (2) a *vitality* motive, as seen in the experience of “existential realities not of *having a body* but of *being a body*… a tactilely, kinesthetically, and affectively attuned body experiencing its own movement” (Sheets-Johnstone, 2024, p. 1), but also (3) an *expressiveness* motive, as seen in the expression of various feelings and experiences in the dance (e.g., Langer, 1957), and (4) an *interpersonal attunement* motive when dancers engage in synchronized expressive movements. As to the latter, Forlè (2024) has argued that good performances of dancing partners are characterized by rhythmic alignment and interpersonal *vitality attunement,* although this does not require any *emotional* attunement between the dancers:

two dancers can be perfectly attuned in the forms of vitality of their movements without this meaning that they are also attuned in corresponding emotional or affective states. A tango can express passionate love even though the dancers barely know each other and are definitely not in love with one another (Forlè, 2024, p. 565).

Just as in the case of physical exercise, we may expect these motives to differ in strength from one individual to another, and from one occasion to another. At one extreme, we may find individuals who work hard to learn the steps in a dance or choreography to perfection, with little attention to how it feels to dance or to the feelings that are expressed, and at the other extreme we may find individuals who prefer to dance spontaneously to the rhythm of the music or focus on the expression of feelings. But, again, it is an empirical question which combination of motives is most clearly associated with wellbeing.

### 3.5 Felt embodiment motivation and self-actualization

One possibility is to see vitality and expressiveness (and other forms of embodiment motivation) as representing an aspect of self-actualization as defined by Maslow (1954). More specifically, the motive to feel one’s body and to express these bodily feelings can be seen as a desire to *actualize one’s experience of being this particular body,* by feeling its vitality and aliveness, and finding ways of bodily expression that are congruent with one’s feelings and with one’s personality. As discussed above, this would imply a focus not only on the *goals* of embodied activities but also on the *ongoing process*. Maslow (1954) points to something similar when he suggests a differentiation “between striving (doing, coping, achieving, trying, purposiveness) and being-becoming (existing, expressing, growing, self-actualization)” (p. 229). Against “a too exclusively means-oriented attitude toward life,” he argues that experiences and behaviors, even though they may be means to some other goal, “can be enjoyed, savored, and appreciated for their own sake” (p. 235), as a kind of “biopleasure, zestful experiencing” (p. 236). Yet, somewhat surprisingly, he characterizes these experiences as “an automatic, unsought-for, unmotivated by-product of being alive and healthy” (p. 236). This is at odds with our notion of felt embodiment *motivation*, as outlined above. The very concept of embodiment motivation implies that experiences of bodily vitality and expressiveness can be a reason for engaging in activities such as physical exercise, dancing and other bodily activities.

### 3.6 Summary of section 3

- Movement is intrinsically connected with feeling; in other words, movement is typically *felt* as movement.
- Evidence indicates that individuals need to feel embodied in movement and physical activity. Felt embodiment can be important not only for one’s wellbeing and health but also for the development of one’s self-identity and interpersonal relations.
- Individuals who engage in physical activity, dancing, and other forms of bodily movement, may do this for various motives. They may differ in the degree to which they are motivated by the felt qualities of their movement, and the degree to which they are moved by other more externally oriented motives.

## 4 Felt embodiment in wholesome environments

In this section, our aim is to discuss some aspects of embodiment motivation in relation to the environment. The argument proceeds in three steps. First, we illustrate what felt embodiment means in relation to our surrounding environment by using visual perception as the main example. Second, we suggest that we also have a *need* to feel embodied in wholesome environments. And third, our discussion turns to the possible role of *motives* to feel embodied in our surroundings.

### 4.1 Felt embodiment as part of the surrounding environment

According to Gibson’s ecological approach to perception (Gibson, 1979) the most important information that is provided via our senses is about the environment’s affordances (see also section 2.1), defined as what the environment affords the individual in terms of possible interactions, for good or bad. For example, an open environment affords locomotion in any direction over the ground; objects of a certain shape and size afford sitting; different kinds of tools afford construction, manipulation, etc. According to Gibson’s theory, and in contrast to many other theories of perception, perception is *not* based on sensation. Still, he admits the essential importance of sensations for the awareness of the *self*. This is seen, for example, in his differentiation between the *visual world* and the *visual field* (Gibson, 1950); although we normally pay attention to the visual world (the things around us with their affordances), we may also shift our attention to the visual field, i.e., how the world *appears* to us from our specific perspective.

The temporary array of perspective appearances of the world is called the field of visual sensations, or the visual field, and this, I think, is the best index an observer has of himself as *here*. So I have to admit that the study of sensations is important for an understanding of one’s awareness of the self even when I deny that it is basic to an understanding of one’s awareness of the world (Gibson, 1969, p. 409).

For some purposes we even *need* to pay attention to how the surrounding environment appears to us from our specific perspective. Painters and photographers, for example, may need to train this kind of attention.

More generally, whichever sensory modality is used, it is possible to shift attention from a focus on the world around us to our perceptual *perspective* on the world. Although the body with its senses is most often a *medium* through which we perceive the world, we can also shift attention *to* this medium by focusing on *how* the world appears to us through our senses. As Gibson formulates it, what we do in the latter case is that we attend to how it *feels* to perceive the world around us. In our terminology, this represents a form of felt embodiment in the surrounding environment.

Two other concepts that are relevant here are affective atmosphere (e.g., Böhme, 2017; Griffero, 2019) and resonance (Rosa, 2019). *Affective atmosphere* is a concept that is used in various areas, including architecture, where it refers to how certain material surroundings feel for the person. As defined by Griffero (2019), a prototypical atmosphere exists whether we perceive it or not—an atmosphere, for example, has the potential to overwhelm us when we encounter it. Griffero also speaks about the value of *pathicity*, defined as the ability to feel the presence of situations and value their affordances, and “to resonate through the felt body in accordance with atmospheres” (Griffero, 2019, p. 426). Rosa’s concept of *resonance* (Rosa, 2019) similarly refers to a way of encountering the world (other people, material things, nature, etc.) which involves being truly touched or moved by that which is encountered. In Rosa’s view, a good life requires experiences of resonance; at the same time, however, he points out that such experiences are *not* controllable or predictable. Rosa contrasts the *resonance mode* with an *instrumental* mode that he sees as typical of modernity, and which involves a motivation to make the world (e.g., technology, wealth) successively more available, attainable and accessible. This instrumental mode is seen to have produced change and growth at an accelerating rate (Rosa, 2013) that is detrimental to our wellbeing.

In the present perspective, flourishing (i.e., a good life) is not only a matter of the person’s mode of relating to the environment. The *characteristics of the environment* are also of utmost importance. This is touched upon in the following section.

### 4.2 The need to feel embodied in wholesome environments

When it comes to our felt embodiment in the environmental surroundings, it is important to bear in mind that environments can differ in how they influence our wellbeing; some environments may even be damaging to our health. Environments differ in how well they fit an individual’s particular embodiment, and individuals of different species may have different needs in terms of what serves as their optimal environment. Research indicates that animals have a need for a life in natural environments rather than in captivity; Asian elephants and polar bears who are kept in captivity, for example, are prone to poor health and breeding difficulties (e.g., Clubb and Mason, 2003). Another widespread observation is that, when milk cows are let out after a winter in the barn, they typically show every sign of enjoying it by running around and kicking their heels, nibbling the fresh grass and exploring the place. Cows on pasture-based systems show better health and lower levels of aggression than those on continuous indoor housing (e.g., Arnott et al., 2017).

As to humans, it has been argued that global urbanization reduces greenery and limits the opportunities for children to gain experiences of nature, with negative consequences for their play, health, and wellbeing (e.g., Hedblom et al., 2024). Haidt (2024) similarly has argued that an environment that affords both physical activity and bodily contact with others is of importance for children’s physical and mental health, which may suffer if children’s environments become too “digital” and they spend too much time online. Empirical research also indicates that experienced embodiment among young adolescents is associated with psychological health, and that low degrees of experienced embodiment predict disordered eating, non-suicidal self-injury, anxiety and depression over and beyond what traditional measures of body dissatisfaction do (Foster et al., 2025; Lundh et al., 2025).

As to adult humans, the importance of positive environmental qualities, particularly those of natural settings, have been emphasized for restorative purposes. According to attention restoration theory (Kaplan, 1995), when an individual suffers from fatigue because they have been over-exposed to attention-demanding tasks, they can recover their attentional capacity by immersing themselves in less demanding physical environments. According to Kaplan, these wholesome environments should optimally require merely effortless attention, while enabling a state of soft fascination, and providing the person with a sense of coherence and wholeness. Importantly, however, he also points out that this is not merely a matter of being *exposed* to potentially health-bringing environments; “the key issue as far as the state of mental fatigue and its recovery are concerned is what happens in the mind. Something in the environment that is not taken in by the mind is unlikely to influence it” (Kaplan, 2001, p. 502). In other words, what is needed for restorative purposes is not just *exposure* to wholesome environments, but the *felt experience* of being an embodied part of these environments. Kaplan (2001) accordingly suggests that the provision of restorative environments be complemented with the acquisition of skills such as “mental resonance, mindfulness, and meditation” (p. 502). This may be described as a need to be in sensuously embodied *contact* with wholesome environments.

### 4.3 Motives of tranquility and connectedness

Motives mentioned for being in embodied contact with nature include not only desires to feel tranquility, peace and harmony, but also to feel connected with something “larger” (e.g., Ashley, 2007; Naor and Mayseless, 2020). As natural environments can afford such experiences of interconnectedness, Naor and Mayseless (2020) speak about “nature as an embodiment of spirituality” (p. 114). Feelings of awe and wonder are often described in this context. Although such feelings are sometimes interpreted in spiritual or religious terms as being in contact with something “sacred” or “divine,” the focus here is on aspects of felt embodiment that are involved, and not on the interpretation of such experiences.

Lundh (2024) describes a mindful embodiment-gratitude practice that was originally developed for a client who expressed a desire for a spiritually oriented nature-focused exercise. The practice was developed to fit an environment where she could see grass and trees, hear birds sing, and feel the wind. The practice had the following self-instructions, with four “verses,” to be learned by heart and to be repeated slowly with a mindful focus on the meaning of each word:

*Explore* how it feels to breathe. Notice the air going in and out. Note how the breathing *connects* me with the surrounding world, makes me inseparable from my surroundings. *Thanks* for the air that surrounds me, the air that I breathe, that gives me life. Thanks for the lungs that make me able to breathe.

*Explore* the sights in front of me, with all the different shades of colour and shapes of things. Note how the light *connects* me with the surrounding world, makes me inseparable from my surroundings. *Thanks* for the light that presents me with all these things, and thanks for my eyes that make me able to see.

*Explore* the sounds around me, the different sounds that come and go. Note how the sounds *connect* me with the surrounding world, make me inseparable from my surroundings. *Thanks* for the air that passes all these sounds to me, and thanks for my ears that make me able to hear.

*Explore* the feelings in my skin and in my whole body. Note all the sensations that *connect* me with my body, make me inseparable from my body. *Thanks* for the body that gives me access to the world in my own particular way (Lundh, 2024, p. 119–120).

This client enjoyed the practice and felt intrinsically motivated to continue using it; this thereby provides an example of motivation to feel embodied in natural surroundings.

Other examples of felt embodiment in the surroundings are not spiritually oriented in the same way. One example is a practice of mindful driving, which was developed for a client who experienced difficulties staying alert while driving long distances. Here the self-instructions involved four phases, each characterized by a different focus of attention: (1) the road; (2) the traffic flow; (3) the landscape; and (4) the sitting (for a detailed description of the self-instructions, see Lundh, 2020, p. 500–501). The practice included instructions to shift between these four phases in accordance with the changing traffic conditions, and to gently bring attention back to one of the phases if attention drifted away. Again, this client felt intrinsically motivated to engage in this practice during driving, and “reported that he could now *enjoy* driving in a new way, as it afforded him new visual and other sensory impressions of the road, the surrounding landscape, and the traffic flow, while sitting comfortably with a straight back behind the wheel and feeling in control of the car’s movement through the environment” (Lundh, 2020, p. 501). This provides an illustration of felt embodiment motivation in an everyday situation.

### 4.4 Summary of section 4

- It is possible to shift attention from a focus on the world around us to our perceptual *perspective* on the world, or how it feels to perceive our surroundings. This provides a kind of felt embodiment in these surroundings.
- Environments differ in how well they fit an individual’s particular embodiment, and individuals of different species may have different needs in terms of what serves as their optimal environment. We have a need to feel embodied as part of wholesome environments.
- Among the motives for being in embodied contact with wholesome environments are not only desires to feel tranquility, peace and harmony, but also to feel oneself as bodily interconnected with the larger environment (motives which are sometimes described as spiritual).

## 5 Felt embodiment in relation to others

The purpose of the following section is to discuss briefly some aspects of embodiment motivation in relation to embodied others. Because this is a huge field that encompasses a large variety of relational experiences, the section can at best provide some sketchy illustrations of some experiences that are at stake. In view of the relative neglect of this field of experience in present research, we think it is important to draw attention to this field’s very existence by illustrating some of its basic phenomena. The section is structured in the same way as in the two preceding sections. That is, the argument proceeds in three steps. First, we provide some examples of felt embodiment in relation to others. Second, we discuss some reasons why we have a *need* to feel embodied in relation to others. And third, we turn the discussion to some *motives* to feel embodied in relation to others.

### 5.1 Felt embodiment in relation to others

This is a field that can be referred to as *embodied relationality* (Lundh and Foster, 2025). It is a largely unexplored area when it comes to its overall structure, although various phenomena within this field have been addressed by some researchers. One differentiation is between (1) the *felt experience* of embodied others and (2) embodied *interactions* with others, with or without direct bodily contact.

#### 5.1.1 The felt experience of embodied others

As we have argued elsewhere (Lundh and Foster, 2025), it is possible to speak about *embodied others* in the same sense as an embodied self, that is, as a passive synthesis:

What characterizes embodied others—just as the embodied self –is not only that they *have* a body (i.e., an objectively perceivable body) but also that they *are* this body (i.e., they *feel* their body subjectively from within); and, moreover, that aspects of these subjective feelings are expressed in movements of their body. The latter makes it possible for us to relate to embodied others *immediately* as a passive synthesis of their “body” and “mind… That is, we perceive embodied others as *expressing* themselves (the body they *are*) in bodily movements (the body they *have*). The crucial thing here is *expressiveness*.

Our felt embodiment is intrinsically connected to *expressions*, as described for example by Langer (1967) in terms of “felt life” and Vendrell Ferran (2021) in terms of feelings of vitality (see also section 2.3). These expressions enable an *intersubjective bridge* between individuals, as the same expressions that we recognize in ourselves are typically also perceivable in others.

The concept of *atmosphere*, which was discussed in section 4.1, is also applicable to interpersonal relations. Roald et al. (2021), for example, applied it in an observational study of mother-infant interactions. To identify atmospheres, they looked for affectivity in the quality of the interacting partners’ movements, facial expressions, vocalizations, gaze, and expressed intentions. They noted that sometimes two completely different atmospheres could surround mother and child, whereas at other times they could observe the development of a *shared* atmosphere. Whether a shared atmosphere developed or not, this illustrates how an observer (in this case the researcher) can perceive an affective atmosphere around another person, and even aspects of the embodied relationality between others.

Such perceptions of an affective atmosphere around others do not typically represent merely some kind of objective registration of that atmosphere, but are typically *felt* by the perceiver. This represents yet another illustration of how bodily feelings and sensations, as discussed above (section 3.1), are ubiquitous in our everyday experience. As embodied creatures, we not only perceive others, but we also *feel* their presence. This is perhaps most obvious when we feel attracted to another person or when we feel repulsion toward someone, but in between these two opposite poles there is a whole spectrum of less conspicuous feelings and sensations attached to the experience of embodied others.

#### 5.1.2 Embodied interactions

The bodily communicative and expressive ties to others when our bodily movements, gestures and comportments are spontaneously coordinated has been called *intercorporeality* (e.g., Merleau-Ponty, 1962). Behnke (2008) speaks about this as a form of *interkinaesthetic affectivity* that is of importance in all kinds of ordinary interpersonal contexts. An ordinary example is our everyday maneuvering in shared social space, which often takes the form of a spontaneous “interkinaesthetic civility” with “a pervasive affective tone that is a little like the flavor of water: seldom thematized unless it tastes ‘wrong,’ yet utterly recognizable” (Behnke, 2008, p. 152). This represents a form of intercorporeality that does not contain much of explicit emotion so long as it flows smoothly, but yet has “a pervasive affective tone.” But intercorporeality can also take the form of more explicit emotional interactions. Fuchs and Koch (2014) describe a model of how social encounters involve “two cycles of embodied affectivity” (p. 5), “a circular interplay of expressions and reactions running in split seconds and constantly modifying each partner’s bodily state” (p. 6) and that “connects both bodies in interbodily resonance or intercorporeality” (p. 6).

Cycles of embodied affectivity of this kind may, of course, also involve direct physical contact. There are, however, also many examples of embodied interactions with direct physical contact that are not primarily emotional. Simple examples are when two individuals are shaking hands, or dancing (see an example from Forlè, 2024 in section 3.4).

### 5.2 The need for embodied contact with significant others

The need to feel in embodied contact with others takes many forms. Most basically, because the survival of the human species is based on sexual reproduction, men and women need to engage in intimate bodily interaction. But bodily contact is needed not only for the survival of the species, but also for the flourishing of individuals; for example, pleasurable sexual interactions may be important for flourishing.

Another illustration of the need for embodied contact comes from Harlow’s cruel experiments on rhesus monkeys, who were separated from their mothers within hours after birth and raised in a cage (Harlow, 1959). Although the cage-raised monkeys survived and appeared healthy, they were unable to develop normal social relations with other monkeys. In one illustrative experiment, Harlow raised eight monkeys in a cage with two surrogate mothers: one made of wire mesh and the other with a layer of foam and then a layer of soft terry-cloth. Even when the bare-wire “mothers” provided them with food and the cloth-covered “mothers” did not, the monkeys overwhelmingly chose contact with the cloth mother and only visited the wire mother when hungry. Harlow concluded that young mammals have a basic need for the *contact comfort* that is typically afforded by the physical contact with their mother; in the absence of a real mother, they may seek out whatever feels most like a mother.

This illustrates how *the qualities of felt contact* with an embodied other may represent an important need for individual flourishing to occur. This may possibly also be generalized to the social level, as when Panksepp (1998) in his textbook on affective neuroscience claims that.

It has long been known that human societies that encourage physical closeness, touching, and the free play of intimacy tend to be the least aggressive in the world (Panksepp, 1998, p. 257).

The importance of close physical contact such as touching has been studied in research on infants (e.g., Hertenstein, 2002). Although there is limited research on the need for intimate physical relations among adults, studies do indicate that touching, hugging and cuddling among persons who are in a romantic relationship is associated with various benefits in terms of health, wellbeing, and relational quality (e.g., van Raalte et al., 2021). The need for skin contact is reflected in some languages that have a separate word for the hunger or longing for it (e.g., “huidhonger” in Dutch).

### 5.3 Embodiment motivation in physical contact with others

In Reiss (2004) taxonomy of motives, the desire for sex represents one of the basic desires, and the associated intrinsic feeling is described as “lust.” Again, although this might represent a rather typical case, there are many variations. Just as in the case of physical activity (see section 3.3), it is possible to differentiate between motives focused on *the end goal* of sexual activity (e.g., orgasm) and motives that involve a focus on loving, sensual, and sexual feelings in *the process* of sexual interaction. Both goal- and process-related motives represent variants of embodiment motivation. It may be expected, however, that pleasure- and love-motivated sexual behavior tends to involve more temporally extended and more thematically diversified forms of felt embodiment than sexual activity engaged in merely for the purpose of achieving orgasm. It is an empirical question to explore the varieties of bodily sensations and emotions that are involved in sexual behavior, and which of these may serve as motives for engaging sexually. As pointed out by Kleinplatz et al. (2022), based on their review of the research in this area, however, the research literature on optimal couple sexual experiences is meager.

There are also important non-sexual varieties of close physical contact, such as touching, hugging, and cuddling. Cuddling has been defined as intimate, physical, and loving contact that involves the whole body to some degree (van Anders et al., 2013). Research, however, shows that individuals differ widely in how comfortable they are to engage in intimate physical interactions. Chopik et al. (2014), for example, found that people high on attachment avoidance were less positive to cuddling in both adult romantic relationships and parent–child relationships. One possible variation here is a form of disembodiment motivation, where the individual avoids physical contact because it has become associated with feelings of discomfort. Importantly, touching, hugging and cuddling may also vary a lot in *felt quality* and the feelings that are expressed. A reasonable hypothesis is that experiences of a loving *touch* as well as loving *touching* are more conducive to flourishing than other varieties of physical contact. In other words, research on intimate physical contact would benefit from much more focus on the expressed and felt *qualities* of the touch—this still seems be a largely unexplored area.

### 5.4 Summary of section 5

- The expressions of the felt body enable an *intersubjective bridge* between individuals, as the same expressions that we recognize in ourselves are typically also perceivable in others.
- As embodied creatures, we not only perceive others; we also *feel* their presence.
- Embodied interactions occur in many different forms, including interkinaesthetic affectivity, vitality attunement and emotional attunement.
- Evidence indicates that individuals have a need for embodied contact with significant others to develop and flourish.
- Although research indicates that the motivation for intimate physical contact shows large individual variation, the study of the *felt quality* of intimate physical contact is a largely unexplored area.

## 6 Conclusion

In the present paper we have outlined a new area of research: *embodiment motivation*, defined as the motivation to feel one’s body (1) “from within,” with its subjectivity, vitality and expressiveness, (2) as embedded in wholesome environments, and (3) in close physical and loving contact with significant others. Our main hypothesis is that the motive to feel embodied in these various contexts is an important element in human flourishing. In this context, we have also suggested that its opposite, *disembodiment motivation* (defined as the motive to avoid or reduce bodily self-experiences, for example due to physical or emotional pain or other forms of bodily discomfort) may be an important element in psychopathology—although, due to space limitations, we have not been able to develop this theme in the present paper.

We consider this area of research to be *transdisciplinary* and based on *methodological pluralism*. Its transdisciplinary nature is seen in the many different disciplines that we have referred to in the present paper. Although the majority of researchers that have been referred have their roots in psychological science, we have also have referred to several philosophers, mostly from the phenomenological tradition (e.g., Husserl, Fuchs, Griffero, Merleau-Ponty, Sheets-Johnstone) but also from other philosophical traditions (e.g., Godfrey-Smith, Langer, Shusterman), as well as scholars from sociology (Rosa) and neuroscience (Damasio, Edelman). As to methodological pluralism, we have referred to research based on experimental, correlational, and phenomenological methods.

As we see it, embodiment motivation is part of a larger field of research which also includes other aspects of embodiment, such as the role of embodiment in self-identity and mental health (e.g., Lundh and Foster, 2024) and embodied relationality (Lundh and Foster, 2025). One task would be to map the field and describe the various *phenomena* in this field. A related task is to analyze how these phenomena should best be *conceptualized*, for the purpose of eventually reaching consensus among researchers about a conceptual system that can form the basis of a paradigm for further research. For example, at present much of the literature in this field is characterized by what may look like a proliferation of different “bodies” (as seen in the distinction between the physical body and the lived, living, or felt body) with risk for a reification of dual realities. One possible way of solving this terminological problem is to use *embodiment* as an overarching concept, thereby aligning with an ontology based on *process* models rather than *substance* models (e.g., Bickhard, 2025; Lundh, 2025), as these are less likely to invite dualistic forms of thinking.

Husserl (1980), in one of his less known remarks, once suggested the development of a discipline of “somatology,” defined as “the science of the animate organism” (p.7), which would include the phenomenological study of bodily experiences. Shusterman (2008) likewise has suggested a new discipline called “somaesthetics.” Both these terms are problematic, as they lie close to already existing physiological terms. Labeling the proposed discipline *embodiment science*, or something similar, might indicate more unambiguously what is at stake.

## Data Availability

The original contributions presented in the study are included in the article/supplementary material, further inquiries can be directed to the corresponding author.

## Footnotes

1 A terminological note: as used here, the term *experienced embodiment* is an overarching term that refers to the synthetic experience of “my body,” including both its objective and subjective aspects, whereas *felt embodiment* refers specifically to the subjective component.

2 Husserl (2001) differentiates between active and passive syntheses. Both involve a combination or unification of aspects of experience, but whereas an *active* synthesis is carried out at a conscious level by means of attention, reflection and judgment, a *passive* synthesis occurs associatively at a pre-reflective level.

3 “Sensings” is an English translation of the German neologism “Empfindnisse,” to differentiate these kinds of lived experiences from mere “Empfindungen” (i.e., sensations).

## References

[ref1] Al-Saji A. (2000). The site of affect in Husserl’s phenomenology: sensations and the constitution of the lived body. Philos. Today 44, 51–59. doi: 10.5840/philtoday200044Supplement6

[ref2] Al-Saji A. (2010). Bodies and sensings: on the use of Husserlian phenomenology for feminist theory. Cont. Philos. Rev. 43, 13–37. doi: 10.1007/s11007-010-9135-8

[ref3] Arnott G. Ferris C. P. O’Connell N. E. (2017). Review: welfare of dairy cows in continuously housed and pasture-based production systems. Animal 11, 261–273. doi: 10.1017/S1751731116001336, PMID: 27364762

[ref4] Ashley P. (2007). Toward an understanding and definition of wilderness spirituality. Aust. Geogr. 38, 53–69. doi: 10.1080/00049180601175865

[ref5] Behnke E. A. (2008). Interkinaesthetic affectivity: a phenomenological approach. Cont. Philos. Rev. 41, 143–161. doi: 10.1007/s11007-008-9074-9

[ref6] Behnke E. A. (2018). On the transformation of the time-drenched body: kinaesthetic capability-consciousness and recalcitrant holding patterns. J. Conscious. Stud. 25, 89–111.

[ref7] Bickhard M. H. (2025). The whole person. Towards a naturalism of minds and persons. London: Elsevier/Academic Press.

[ref8] Böhme G. (2017). Atmospheric architectures. The aesthetics of felt spaces. London: Bloomsbury.

[ref9] Chopik W. J. Edelstein R. S. van Anders S. M. Wardecker B. M. Shipman E. L. Samples-Steele C. R. (2014). Too close for comfort? Adult attachment and cuddling in romantic and parent–child relationships. Pers. Individ. Differ. 69, 212–216. doi: 10.1016/j.paid.2014.05.035

[ref10] Clubb R. Mason G. (2003). Captivity effects on wide-ranging carnivores. Nature 425, 473–474. doi: 10.1038/425473a, PMID: 14523435

[ref11] Crum A. J. Langer E. J. (2007). Mind-set matters. Exercise and the placebo effect. Psychol. Sci. 18, 165–171. doi: 10.1111/j.1467-9280.2007.01867.x, PMID: 17425538

[ref12] Damasio A. (2012). Self comes to mind. London: Vintage.

[ref13] Damasio A. (2018). The strange order of things. Life, feeling, and the making of cultures. London: Robinson.

[ref14] Damasio A. (2021). Feeling and knowing. Making minds conscious. London: Robinson. 10.1080/17588928.2020.1846027 33323038

[ref15] Deci E. L. Ryan R. M. (2000). The “what” and “why” of goal pursuits: human needs and the self-determination of behavior. Psychol. Inq. 11, 227–268. doi: 10.1207/S15327965PLI1104_01

[ref16] Denton D. (2006). The primordial emotions. Oxford scholarships online. Oxford: Oxford University Press.

[ref17] Edelman G. M. (1989). The remembered present: a biological theory of consciousness. New York: Basic Books.

[ref18] Edelman G. M. (1992). Bright air, brilliant fire: on the matter of the mind. London: Basic Books.

[ref19] Ferrarello S. (2024). Affect disorders: an Husserlian interpretation of alexytimia, BPD and narcissistic traits. Hum. Stud. 47, 535–551. doi: 10.1007/s10746-024-09712-x

[ref20] Forlè F. (2019). From the embodied self to the embodied person. On the constitution of one’s own personal expressive style. Hum. Mente J. Philosophical Stud. 12, 100–120.

[ref21] Forlè F. (2024). The sense of we-agency and vitality attunement: between rhythmic alignment and emotional attunement. Phenomenol. Cogn. Sci. 23, 551–571. doi: 10.1007/s11097-021-09779-2

[ref22] Foster L. Lundh L. G. Daukantaité D. (2025). Embodiment and psychological health in adolescence. 1. Development and validation of a brief 12-item questionnaire to measure the experience of embodiment. J. Pers. Oriented Res. 11, 10–24. doi: 10.17505/jpor.2025.27576, PMID: 40207190 PMC11977780

[ref23] Frankl V. E. (1963). Man’s search for meaning. New York: Washington Square Press.

[ref24] Fuchs T. (2022). The disappearing body: anorexia as a conflict of embodiment. Eat. Weight Disord. 27, 109–117. doi: 10.1007/s40519-021-01122-7, PMID: 33666885 PMC8860785

[ref25] Fuchs T. Koch S. (2014). Embodied affectivity: on moving and being moved. Front. Psychol. 5:508. doi: 10.3389/fpsyg.2014.00508, PMID: 24936191 PMC4047516

[ref26] Gallup G. G. Jr. Anderson J. R. (2020). Self-recognition in animals: where do we stand 50 years later? Lessons from cleaner wrasse and other species. Psychol. Conscious. Theory Res. Pract. 7, 46–58. doi: 10.1037/cns0000206

[ref27] Gibson J. J. (1950). The perception of the visual world. Boston: Houghton Mifflin.

[ref28] Gibson J. J. (1966). The senses considered as perceptual systems. Boston: Houghton Mifflin.

[ref29] Gibson J. J. (1969). Are there sensory qualities of objects? Synthese 19, 408–409. doi: 10.1007/BF00485657

[ref30] Gibson J. J. (1979). The ecological approach to visual perception. Boston: Houghton Mifflin.

[ref31] Godfrey-Smith P. (2017). Other minds: the octopus, the sea, and the deep origins of consciousness. New York: Harper-Collins.

[ref32] Griffero T. (2019). Pathicity: experiencing the world in an atmospheric way. Open Philos. 2, 414–427. doi: 10.1515/opphil-2019-0031

[ref33] Haidt J. (2024). The anxious generation. New York: Allen Lane.

[ref34] Harlow H. F. (1959). Love in infant monkeys. Sci. Am. 200, 68–74. doi: 10.1038/scientificamerican0659-68, PMID: 13658993

[ref35] Hedblom M. Mårtensson F. Sang Å. O. Wiström B. Litsmark A. (2024). Play biotopes put into practice—creating synergies between children and nature. People Nat. 6, 2046–2059. doi: 10.1002/pan3.10708

[ref36] Heider F. Simmel M. (1944). An experimental study of apparent behavior. Am. J. Psychol. 57, 243–259. doi: 10.2307/1416950

[ref37] Heinämaa S. (2018). “Embodiment and bodily becoming” in The Oxford handbook of the history of phenomenology. ed. Zahavi D. (Oxford: Oxford University Press).

[ref38] Hertenstein M. J. (2002). Touch: its communicative functions in infancy. Hum. Dev. 45, 70–94. doi: 10.1159/000048154

[ref39] Husserl E. (1970). The crisis of the European sciences and transcendental phenomenology. Evanston: Northwestern University Press.

[ref40] Husserl E. (1980). Phenomenology and the foundation of the sciences. The Hague: Martinus Nijhoff Publishers.

[ref41] Husserl E. (1989). Ideas pertaining to a pure phenomenology and to a phenomenological philosophy. Second book: studies in the phenomenological constitution. Dordrecht: Kluwer.

[ref42] Husserl E. (2001). Analyses concerning passive and active synthesis. Lectures on transcendental logic. Dordrecht: Kluwer Academic Publishers.

[ref43] Kabat-Zinn J. (2013). Full catastrophe living: using the wisdom of your body and mind to face stress, pain and illness. Revised and updated edition. New York: Bantam Books.

[ref44] Kaplan S. (1995). The restorative benefits of nature: toward an integrative framework. J. Environ. Psychol. 15, 169–182. doi: 10.1016/0272-4944(95)90001-2

[ref45] Kaplan S. (2001). Meditation, restoration, and the management of mental fatigue. Environ. Behav. 33, 480–506. doi: 10.1177/00139160121973106

[ref46] Kee H. (2023). Phenomenological insights from postural yoga practice. In Ferrarello S. & and Hadjioannou C. (Eds.), The Routledge handbook of phenomenology of mindfulness Cham, Switzerland: Springer (pp. 138–151).

[ref47] Kleinplatz P. J. Charest M. Rosen L. A. Ménard A. D. (2022). Optimal couple sexuality: a review of the (limited) literature. Curr. Sex. Health Rep. 14, 63–69. doi: 10.1007/s11930-022-00327-w

[ref48] Langer S. K. (1957). Problems of art. New York: Scribner.

[ref49] Langer S. K. (1967). Mind: an essay on human feeling, vol. 1 Baltimore: Johns Hopkins University Press.

[ref50] Larkey L. James D. Vizcaino M. Kim S. W. (2024). Effects of tai chi and Qigong on heart rate variability: a systematic review and meta-analysis. Heart and Mind 8, 310–324. doi: 10.4103/hm.HM-D-24-00045 37695835

[ref51] Lo W. Y. Liu X. Cheung D. S. Lin C. C. (2025). Effects of tai chi and Qigong in depressive and anxiety symptoms in cancer patients: an overview of systematic reviews. Heart Mind 9, 136–146. doi: 10.4103/hm.HM-D-24-00090

[ref52] Lundh L. G. (2020). Experimental phenomenology in mindfulness research. Mindfulness 11, 493–506. doi: 10.1007/s12671-019-01274-9

[ref53] Lundh L. G. (2022a). Experimental phenomenology as personalized health intervention: a case illustration. OBM Int. Complement. Med. 7:11. doi: 10.21926/obm.icm.2201008

[ref54] Lundh L. G. (2022b). Experimental phenomenology as an approach to the study of contemplative practices. Front. Psychol., 12:751298. doi: 10.3389/fpsyg.2021.751298, PMID: 35082715 PMC8784732

[ref55] Lundh L. G. (2024). “Experimental phenomenology in research on spirituality” in The Routledge international handbook of research methods in Spirituality & Contemplative Studies. eds. Clough K. Flanagan B. (New York: Routledge), 113–121.

[ref56] Lundh L. G. (2025). Book review: “the whole person. Towards a naturalism of minds and persons” by mark H. Bickhard. J. Pers. Oriented Res. 11, 107–113. doi: 10.17505/jpor.2025.28096

[ref57] Lundh L. G. Foster L. (2024). Embodiment as a synthesis of having a body and being a body, and its role in self-identity and mental health. New Ideas Psychol. 74:101083. doi: 10.1016/j.newideapsych.2024.101083

[ref58] Lundh L. G. Foster L. (2025). The ethics of relating to patients as embodied others. In Ferrarello Susi (Ed.), Handbook of phenomenological bioethics. Bloomsbury. In press.

[ref59] Lundh L. G. Foster L. Daukantaitė D. (2025). Embodiment and psychological health in adolescence. 2. Embodiment profiles and their association with psychological health problems among young adolescents. J. Pers. Oriented Res. 11, 25–35. doi: 10.17505/jpor.2025.27578 40207191 PMC11977779

[ref60] Maiese M. (2024). Anorexia nervosa, bodily alienation, and authenticity. Rev. Philos. Psychol. 15, 773–793. doi: 10.1007/s13164-023-00717-6, PMID: 41215982

[ref61] Maslow A. H. (1954). Motivation and personality. New York: Harper and Row.

[ref62] Merleau-Ponty M. (1962). Phenomenology of perception. London: Routledge and Kegan.

[ref63] Murray H. A. (1938). Explorations in personality. New York: Oxford University Press.

[ref64] Naor L. Mayseless O. (2020). The therapeutic value of experiencing spirituality in nature. Spirit. Clin. Pract. 7, 114–133. doi: 10.1037/scp0000204

[ref65] Panksepp J. (1998). Affective neuroscience. The foundations of human and animal emotions. Oxford: Oxford University Press.

[ref66] Panksepp J. (2016). The cross-mammalian neurophenomenology of primal emotional affects: from animal feelings to human therapeutics. J. Comp. Neurol. 524, 1624–1635. doi: 10.1002/cne.23969, PMID: 26876723

[ref67] Reiss S. (2004). Multifaceted nature of intrinsic motivation: the theory of 16 basic desires. Rev. Gen. Psychol. 8, 179–193. doi: 10.1037/1089-2680.8.3.179

[ref68] Reiss S. Havercamp S. M. (1998). Toward a comprehensive assessment of fundamental motivation: factor structure of the Reiss profile. Psychol. Assess. 10, 97–106. doi: 10.1037/1040-3590.10.2.97

[ref69] Reiss S. Wiltz J. Sherman M. (2001). Trait motivational correlates of athleticism. Pers. Individ. Differ. 30, 1139–1145. doi: 10.1016/S0191-8869(00)00098-2

[ref70] Roald T. Boldsen S. Køppe S. (2023). Movement and meaning. An investigation in early intersubjective development. Metodo 11, 315–354. doi: 10.19079/metodo.11.2.315

[ref71] Roald T. Pedersen I. E. Levin K. Køppe S. (2021). Atmospheric infancy. Qual. Psychol. 8, 311–327. doi: 10.1037/qup0000121

[ref72] Rosa H. (2013). Social acceleration. A new theory of modernity. New York: Columbia University Press.

[ref73] Rosa H. (2019). Resonance: a sociology of our relationship with the world. Cambridge, UK: Polity Press.

[ref74] Ryan R. M. Frederick C. (1997). On energy, personality, and health: subjective vitality as a dynamic reflection of well-being. J. Pers. 65, 529–565. doi: 10.1111/j.1467-6494.1997.tb00326.x, PMID: 9327588

[ref75] Ryff C. D. (1989). Happiness is everything, or is it? Explorations on the meaning of psychological well-being. J. Pers. Soc. Psychol. 57, 1069–1081. doi: 10.1037/0022-3514.57.6.1069

[ref76] Schleim S. (2014). Whose well-being? Common conceptions and misconceptions in the enhancement debate. Front. Syst. Neurosci. 8:148. doi: 10.3389/fnsys.2014.00148, PMID: 25191232 PMC4137240

[ref77] Shapiro S. L. Carlson L. E. (2017). The art and science of mindfulness: integrating mindfulness into psychology and the helping professions. Washington, DC: American Psychological Association.

[ref78] Sheets-Johnstone M. (2015). Embodiment on trial: a phenomenological investigation. Cont. Philos. Rev. 48, 23–39. doi: 10.1007/s11007-014-9315-z

[ref79] Sheets-Johnstone M. (2020). The body subject: being true to the truths of experience. J. Specul. Philos. 34, 1–29. doi: 10.5325/jspecphil.34.1.0001

[ref80] Sheets-Johnstone M. (2024). The existential realities of dancing. Front. Cogn. 3:1372945. doi: 10.3389/fcogn.2024.1372945 PMC13281208 42339405

[ref81] Shusterman R. (2008). Body consciousness: a philosophy of mindfulness and somaesthetics: Cambridge University Press.

[ref82] Stults-Kolehmainen M. A. (2023). Humans have a basic physical and psychological need to move the body: physical activity as a primary drive. Front. Psychol. 14:1134049. doi: 10.3389/fpsyg.2023.1134049, PMID: 37113126 PMC10128862

[ref83] Thompson E. (2007). Mind in life: biology, phenomenology, and the sciences of mind. Cambridge, Mass: Harvard University Press.

[ref84] Thompson E. (2022). Could all life be sentient? J. Conscious. Stud. 29, 229–265. doi: 10.53765/20512201.29.3.229

[ref85] van Anders S. M. Edelstein R. S. Wade R. M. Samples-Steele C. R. (2013). Descriptive experiences and sexual vs. nurturant aspects of cuddling between adult romantic partners. Arch. Sex. Behav. 42, 553–560. doi: 10.1007/s10508-012-0014-8, PMID: 23070529

[ref86] van Raalte L. J. Floyd K. Mongeau P. A. (2021). The effects of cuddling on relational quality for married couples: a longitudinal investigation. West. J. Commun. 85, 61–82. doi: 10.1080/10570314.2019.1667021

[ref87] Varela F. J. Thompson E. Rosch E. (1991). The embodied mind: cognitive science and human experience. Cambridge, Mass: MIT Press.

[ref88] Vendrell Ferran I. (2021). “How to understand feelings of vitality: an approach to their nature, varieties, and functions” in Phenomenology of bioethics: technoethics and lived-experience. ed. Ferrarello S., vol. 84, 115–129. Cham, Switzerland: Springer The International Library of Bioethics.

